# Serum levels of IL-4, IL-13 and IL-33 in patients with age-related macular degeneration and myeloproliferative neoplasms

**DOI:** 10.1038/s41598-023-31078-w

**Published:** 2023-03-11

**Authors:** Kathrine Gotfredsen, Charlotte Liisborg, Vibe Skov, Lasse Kjær, Hans Carl Hasselbalch, Torben Lykke Sørensen

**Affiliations:** 1 The Department of Ophthalmology, Zealand University Hospital, Roskilde, Denmark; 2 The Department of Hematology, Zealand University Hospital, Roskilde, Denmark

**Keywords:** Eye diseases, Haematological diseases

## Abstract

Immune responses play a key role in the pathogenesis and progression of myeloproliferative neoplasms (MPN) and age-related macular degeneration (AMD). Recent studies suggested using MPNs as a “Human Inflammation Model” of drusen development and previous results showed interleukin-4 (IL-4) dysregulation in MPN and AMD. IL-4, IL-13 and IL-33 are all cytokines involved in the type 2 inflammatory response. This study investigated the cytokine levels of IL-4, IL-13 and IL-33 in serum of MPN and AMD patients. This cross-sectional study included 35 patients with MPN with drusen (MPNd) and 27 with MPN and normal retinas (MPNn), 28 patients with intermediate AMD (iAMD) and 29 with neovascular AMD (nAMD). With immunoassays, we quantified and compared levels of IL-4, IL-13 and IL-33 in serum between the groups. The study was conducted at Zealand University Hospital, Roskilde, Denmark, between July 2018 and November 2020. The serum levels of IL-4 were significantly higher in the MPNd group than in the MPNn group (*p* = 0.003). In regard to IL-33, the difference between MPNd and MPNn was not significant (*p* = 0.069), however, when subdivided into subgroups, a significant difference was found between polycythemia vera patients with drusen and those without drusen (*p* = 0.005). We found no IL-13 difference between the MPNd and MPNn groups. Our data didn’t show any significant IL-4 or IL-13 serum level difference between the MPNd and iAMD groups but in regard to IL-33, data recorded a significant serum level difference between the two groups. There was no statistically significant difference between the MPNn, iAMD and nAMD groups in levels of IL-4, IL-13 and IL-33. These findings suggested that the serum levels of IL-4 and IL-33 might play a role in drusen development in MPN patients. The results might represent the type 2 inflammatory arm of the disease. The findings support the association between chronic inflammation and drusen.

## Introduction

Age-related macular degeneration (AMD) is the leading cause of irreversible central vision loss in developed countries^1^. The early and intermediate stages of AMD are characterized by deposits of lipids and proteins (drusen) between the retinal pigment epithelium (RPE) and Bruch’s membrane (BM). Drusen are associated with the degeneration of the RPE which leads to a dysfunction or loss of the photoreceptors. Pigmentary abnormalities also occur in the intermediate stage. Late-stage AMD is characterized by choroidal neovascularization (neovascular AMD, nAMD) and/or geographic atrophy (GA)^2^. AMD is associated with aging but the exact pathogenesis of AMD and the reasons why drusen are develop are not entirely understood^3^. Increasing evidence suggests a role of chronic inflammation in development and progression of AMD^4,5^.

The Philadelphia-negative chronic myeloproliferative neoplasms (MPNs) are clonal hematopoietic stem cell diseases. These neoplasms include essential thrombocythemia (ET), polycythemia vera (PV), and myelofibrosis (MF). Transitions between the diseases are common, which may depict a biologic continuum from the early disease states (ET/PV) to the more advanced disease state (MF). Recently, the MPNs have been described as a “Human inflammation Model”, implying chronic inflammation as the driving force for premature atherosclerosis development, clonal evolution and an increased risk of second cancer in MPNs. In this perspective, dampening chronic inflammation, driving the malignant clone, together with stem-cell targeting therapy with interferon-alpha2, are novel therapeutic approaches in MPNs^6–11^.

Patients with MPNs are at increased risk of AMD. Studies suggest that this association may be explained by chronic systemic inflammation^12–14^. Chronic inflammation is usually of low grade and persistent and is closely related to aging in both MPN^15^ and AMD^16,17^. The development of low-grade systemic inflammation in aging can be termed “inflammaging”^18^ and this term can help to clarify the association between MPN and AMD. Recent work proposes to use MPNs as a “Human Inflammation Model” of drusen development where chronic systemic inflammation in MPN elicits drusen formation^14,17^.

Interleukin-4 (IL-4) is a signature cytokine of the type 2 inflammatory response. Previous studies have shown IL-4 dysregulation in early and late stages of AMD^19–21^ as well as in patients with ET and PV, in whom higher plasma levels of IL-4 have been found compared to healthy subjects^22^. These findings may implicate IL-4 to play a role in the AMD and MPN physiopathology.

IL-33, a cytokine of the IL-1 family, is a ligand for the IL-1 receptor-related protein ST2 (IL1RLI/ST2). It is known that IL-33 through interaction with ST2 receptor activates Th2 and mast cells, which attract a variety of cytokines, including IL-4 and IL-13^23^. The IL-33/ST2 axis appears to play a pivotal role in Th2-driven chronic inflammatory diseases, such as asthma, inflammatory bowel disease, and allergic rhinitis^24–27^.

In this study we further investigate chronic systemic inflammation in MPN and AMD with focus on the type 2 inflammatory response by measuring and comparing IL-4, IL-13 and IL-33 levels in serum. Initially, we compare the inflammatory cytokines in MPN patients with drusen (MPNd) and MPN patients without drusen (MPNn) to investigate if the cytokines play a role in drusen development in MPN patients. Subsequently, we want to elucidate if the MPN “Human Inflammation Model” of drusen development may be a model of patients with intermediate AMD (iAMD) by measuring possible differences in serum levels of cytokines between the MPNd and iAMD. To unravel possible cytokine differences or similarities between these two groups are interesting in the prevention of the development from iAMD to nAMD. Finally, we compare the type 2 inflammatory cytokines in MPNd, MPNn, iAMD and patients with neovascular AMD (nAMD) because previous studies have shown cytokine dysregulation in both early and late stages of AMD. These comparative studies provide a unique opportunity to further investigate drusen pathophysiology.

## Results

### Study population

We included 119 patients in the study: 35 MPNd, 27 MPNn, 28 iAMD and 29 nAMD. Baseline patient characteristics are summarized in Table 1. The patients are the same as in previous work, and patient characteristics are therefore identical^14^.

**Table 1:** Patient characteristics.

	MPNd (n = 35)	MPNn (n = 27)	iAMD (n = 28)	nAMD (n = 29)	*p* value
**Demographics**					
Age, years, median (IQR)	72 (65–76)	69 (62–74)	73 (68–76)	77 (71–82)	**< 0.001**^a^
Sex					0.28^b^
Males, n (%)	20 (57)	10 (37)	10 (36)	12 (41)	
Females, n (%)	15 (43)	17 (63)	18 (64)	17 (59)	
**Lifestyle factors**					
Smoking, n (%)					0.83^c^
Never	16 (46)	11 (41)	12 (43)	12 (41)	
Former	18 (51)	14 (52)	13 (46)	13 (45)	
Current	1 (3)	2 (7)	3 (11)	4 (14)	
Body mass index (95%CI)	25 (24–27)	27 (25–29)	25 (24–27)	26 (24–27)	0.48^d^
Alcohol consumption, units per week (median (IQR)	7 (2–14)	2 (0–8)	3 (0–7)	2 (0–7)	**0.0036**^a^
**Comorbidities**					
Cardiovascular disease, n (%)	6 (17)	6 (22)	5 (18)	4 (14)	0.89^c^
Hypertension, n (%)	18 (51)	17 (63)	8 (29)	13 (45)	0.075^b^
Hypercholesterolaemia, n (%)	3 (9)	2 (7)	2 (7)	4 (14)	0.82^c^
Type 2 diabetes, n (%)	2 (6)	0 (0)	1 (4)	2 (7)	0.76^c^
**MPN diagnosis (only MPN patients)**			–	–	0.082^b^
Essential thrombocythemia, n (%)	6 (17)	11 (41)	–	–	
Polycythemia vera, n (%)	26 (74)	13 (48)	–	–	
Pre-PMF	0 (0)	1 (4)	–	–	
Primary myelofibrosis, n (%)	3 (9)	2 (7)	–	–	
**Mutation status (only MPN patients)**			–	–	0.43^c^
JAK2V617F, n (%)	31 (91)	22 (82)	–	–	
CALR mutation, n (%)	1 (3)	3 (11)	–	–	
MPL mutation, n (%)	1 (3)	0 (0)	–	–	
Triple-Negative, n (%)	1 (3)	2 (7)	–	–	
**JAK2V617F allele burden**			–	–	0.61^c^
1–25%, n (%)	15 (47)	14 (67)	–	–	
> 25–50%, n (%)	8 (25)	3 (14)	–	–	
> 50–75%, n (%)	7 (22)	3 (14)	–	–	
> 75%, n (%)	2 (6)	1 (5)	–	–	
**Overall JAK2V617F allele burden%, median (IQR) (only MPN patients)**	33 (11–56)	17 (6–28)	–	–	0.089^a^

*AMD* age-related macular degeneration, *nAMD* neovascular AMD, *iAMD* intermediate AMD, *MPN* myeloproliferative neoplasms, *MPNd* patients with MPN and drusen, *MPNn* patients with MPN and normal retinas, *IQR* interquartile range, *95% CI* 95% confidence interval, *Pre-MF* pre-myelofibrosis, *JAK2V617F* mutation in the *JAK2* gene, *CALR* calreticulin gene, *MPL MPL* gene, the gene encoding the thrombopoietin receptor.

Significant *p* values are shown in bold. Statistical comparisons between groups:

^a^Kruskal Wallis test.

^b^Pearson’s Chi-squared test.

^c^Fischer’s exact test.

^d^One-way ANOVA.

Patients with nAMD had a median age of 77 (IQR: 71–82) years, significantly older than iAMD (73 years, IQR: 68–76, *p* = 0,034), MPNd (72 years, IQR 65–76, *p* = 0.0040) and MPNn (69 years, IQR: 62–74, *p* < 0.001). The MPNd group had a significantly higher alcohol consumption in units per week than the other groups (nAMD, *p* < 0.001; iAMD, *p* = 0.019; MPNn, *p* = 0.021). There was no difference in sex, smoking, body mass index and comorbidities. Of the MPN patients, 17 had ET, 39 had PV, 1 had Pre-PMF and 5 had PMF. Most patients with MPN had the *JAK2V617F*-mutation (MPNd, 91%; MPNn 82%)), and fewer had mutations in the calreticulin (*CALR*) gene (MPNd, 3%; MPNn 11%) and the thrombopoietin receptor (*MPL*) gene (MPNd 3%; MPNn 0%). No difference in allele burden between the two MPN groups was detected (*p* = 0.62). The patients with MPNs were all receiving acetylsalicylic acid or other anticoagulant therapy. 27 MPNd and 20 MPNn were receiving hydroxyurea (HU). Distribution in HU treated versus non-HU treated was similar in the two groups (*p* = 0.98). Patients receiving statins were similar across all groups (*p* = 0.58).

### The inflammatory cytokines IL-4, IL-13 and IL-33 in MPN groups

Since it is interesting to investigate the role of the type 2 inflammatory response in the development of drusen in MPN patients, we compared IL-4, IL-13 and IL-33 serum levels in MPNd and MPNn (Table 2). The level of IL-4 in the MPNd group was 0.32 pg/mL compared to 0.29 pg/mL for the MPNn group. The difference was significant (*p* = 0.003). The difference between the MPNd (1.33 pg/mL) and MPNn (1.28 pg/mL) groups in regard to IL-33 was not significant (*p* = 0.069). No statistically significant difference in levels of IL-13 between the MPNd (1.47 pg/mL) and MPNn (1.19 pg/mL) groups was found (*p* = 0.173).

**Table 2:** Levels of the inflammatory cytokines IL-4, IL-13 and IL-33 and comparisons between groups.

Inflammatory cytokines (serum)	MPNd (n = 35)	MPNn (n = 27)	iAMD (n = 28)	nAMD (n = 29)	*p*-value MPNd versus MPNn	*p*-value iAMD versus MPNd	*p*-value nAMD versus iAMD	*p*-value nAMD versus MPNd	*p*-value nAMD versus MPNn	*p-*value iAMD versus MPNn	*p*-value between all groups
IL-4 pg/mL, median (IQR)	0.32 (0.30–0.35)	0.29 (0.28–0.31)	0.30 (0.27–0.34)	0.28 (0.27–0.30)	**0.003**^a^	0.052^a^	0.113^a^	** < 0.001**^a^	0.112^a^	0.60^a^	** < 0.001**^b^
IL-13 pg/mL, median (IQR)	1.47 (0.91–3.04)	1.19 (0.86–1.57)	1.31 (0.97–1.86)	0.85 (0.62–1.60)	0.173^a^	0.507^a^	0.151^a^	0.022^a^	0.384^a^	0.427^a^	0.11^b^
IL-33 pg/mL, median (IQR)	1.33 (1.26–1.45)	1.28 (1.20–1.38)	1.25 (1.14–1.34)	1.30 (1.20–1.38)	0.069^a^	**0.005**^a^	0.179^a^	0.083^a^	0.871^a^	0.173^a^	**0.03**^b^

*IL* interleukins, *IQR* interquartile range, *AMD* age-related macular degeneration, *nAMD* neovascular AMD, *iAMD* intermediate AMD, *MPN* myeloproliferative neoplasms, *MPNd* patients with MPN and drusen, *MPNn* patients with MPN and normal retinas.

*p* values in bold are significant. Statistical test for comparisons:

^a^Wilcoxon rank-sum test.

^b^Kruskal Wallis test.

### Inflammatory cytokines and MPN subgroups

To compare cytokines in MPN subgroups, we divided the MPN patients into ET, PV and PMF (Table 3). There was no significant difference in levels of IL-4 between ET and PV (*p* = 0.305), PV and PMF (*p* = 0.708) or ET and PMF (*p* = 0.865), in levels of IL-13 between ET and PV (*p* = 0.9), PV and PMF (*p* = 0.61) or ET and PMF (*p* = 0.871) and in levels of IL-33 between ET and PV (*p* = 0.958), PV and PMF (*p* = 0.757) or ET and PMF (*p* = 0.973).

**Table 3:** Levels of the inflammatory cytokines IL-4, IL-13 and IL-33 in MPN subgroups regardless of signs of AMD and comparisons between groups.

Inflammatory cytokines (serum)	ET (n = 17)	PV (n = 39)	PMF (n = 6)	*p* value ET versus PV	*p*-value PV versus PMF	*p* value ET versus PMF
IL-4 pg/mL, median (IQR)	0.30 (0.28–0.32)	0.31 (0.28–0.34)	0.29 (0.29–0.33)	0.305	0.708	0.865
IL-13 pg/mL, median (IQR)	1.22 (0.93–1.72)	1.45 (0.85–2.71)	1.20 (0.98–1.41)	0.9	0.61	0.871
IL-33 pg/mL, median (IQR)	1.28 (1.25–1.42)	1.30 (1.24–1.44)	1.36 (1.29–1.39)	0.958	0.757	0.973

*IL* interleukins, *IQR* interquartile range, *ET* essential thrombocythemia, *PV* polycythemia vera, *PMF* primary myelofibrosis.

Statistical comparisons between groups:

^a^Wiloxons rank-sum test.

We further looked at the cytokines in subdivisions of the PV group (Table 4). The PV group was subdivided into 26 PV patients with drusen (PVd) and 13 with normal retinas (PVn). The level of IL-4 in the PVd group was 0.32 pg/mL compared to 0.28 pg/mL for the PVn group (*p* = 0.006). In regard to IL-33, the level was 1.34 pg/mL in the PVd group compared to 1.21 pg/mL in the PVn group, which was also significantly higher (*p* = 0.005). We did not observe the same differences in IL-4 and IL-33 in ET patients when divided in ETd (n = 6) and ETn (n = 11) (Table 4) but this group was small. No difference was seen between the PVd and PVn and ETd and ETn in regard to IL-13.

**Table 4:** Levels of the inflammatory cytokines IL-4, IL-13 and IL-33 in subdivisions of the PV and ET group into those with drusen and those with normal retinas.

Inflammatory cytokines (serum)	PVd(n = 26)	PVn (n = 13)	*p* value PVd versus PVn	ETd (n = 6)	ETn (n = 11)	*p* value ETd versus ETn
IL-4 pg/mL, median (IQR)	0.32 (0.30–0.37)	0.28 (0.27–0.31)	**0.006**	0.30 (0.27–0.32)	0.30 (0.29–0.32)	0.808
IL-13 pg/mL, median (IQR)	1.48 (0.95–2.98)	1.02 (0.71–1.49)	0.119	2.07 (0.88–5.70)	1.22 (0.96–1.55)	0.711
IL-33 pg/mL, median (IQR)	1.34 (1.27–1.46)	1.21 (1.14–1.32)	**0.005**	1.25 (1.25–1.42)	1.34 (1.27–1.41)	0.661

*PV* polycythemia vera, *PVd* patients with PV and drusen, *PVn* patients with PV and normal retinas, *ET* essential thrombocythemia, *ETd* patients with ET and drusen, *ETn* patients with ET and normal retinas, *IL* interleukins, *IQR* interquartile range.

P-values in bold are significant. Statistical test for comparisons:

^a^Wilcoxon rank-sum test.

### The inflammatory cytokines IL-4, IL-13 and IL-33 in the MPNd and iAMD groups

We wanted to investigate possible differences in serum levels between the MPNd and iAMD (Table 2). The IL-4 level in the MPNd group was 0.32 pg/mL compared to 0.30 pg/mL in the iAMD group (*p* = 0.052), which was not statistically significant. Comparing the serum level of IL-13 in the MPNd group (1.47 pg/mL) with the iAMD group (1.31 pg/mL), the difference was not significant either (*p* = 0.507).The level of IL-33 in the MPNd group was 1.33 pg/mL compared to 1.25 pg/mL in the iAMD group. The higher level of IL-33 in the MPNd group compared to the iAMD group was significant (*p* = 0.005).

### The inflammatory cytokines IL-4, IL-13 and IL-33 and comparisons between all groups

Since previous studies showed IL-4 dysregulation in MPN patients and early and late stages of AMD patients, we compared serum cytokine levels between MPNd, MPNn, iAMD and nAMD (Table 2). The IL-4 level in the MPNd group (0.32 pg/mL) was significantly higher than in the nAMD group (0.28 pg/mL, *p* =  < 0.001). There was no statistically significant difference between the MPNn, iAMD and nAMD groups in level of IL-4, IL-13 and IL-33.

## Discussion

In this study, we have for the first time investigated the serum levels of IL-4, IL-13 and IL-33 in patients with MPNd, MPNn, iAMD and nAMD. The IL-4 cytokine was interesting to investigate, because circulating IL-4 levels have been reported to be elevated in both patients with MPN and AMD^19–22^.

Highly interesting, we find that patients with MPNd have a significantly higher level of IL-4 compared to MPNn. Also, the subgroup of PV patients with drusen has a significantly higher level of IL-4 compared to those with PV and normal retinas.

Our results support IL-4 to be involved in the pathogenesis of AMD. Thus, a study with dry and exudative AMD patients and matched controls without AMD found that genetic polymorphisms in IL-4 -590 and intron 3 VNTR, which are reported to affect the production of IL-4, were associated with increased risk of AMD^19^. Sasaki et al. found significantly higher elevations of IL-23, IL-4, and IL-10 in the aqueous humour of patients with polypoidal choroidal vasculopathy (PCV) compared to controls without any retinal diseases. PCV is considered to be one form of AMD but has a distinct morphology, course of progression, and responsiveness to photodynamic therapy^20^. Another study showed that the production of IL-4 and IFN-γ in peripheral blood mononuclear cells (PBMCs) after stimulation with phytohaemagglutinin (PHA) were higher in PCV and nAMD patients than in healthy controls. The same study suggested that circulating IFN-γ and IL-4 producing Th1 and Th2 cells might be involved in the pathogenesis of nAMD^21^.

In our study, no significant differences in IL-4 serum levels between the MPNd and iAMD were recorded. The levels of IL-4 in the nAMD, iAMD and MPNn groups were virtually the same. Together with the above presented findings and previous studies of the increased levels of IL-4 in AMD compared to healthy controls, our findings might indicate that all the MPN and AMD groups had serum concentrations of IL-4, which are elevated compared to healthy controls.

In AMD, the bone marrow supplies macrophages and new vascular endothelial cells to the retina. Analyses of bone marrow cells and chimeric mice suggest that damages of the retina and choroidal tissue release signals to the bone marrow to repair the vascular damage. This signal induces a recruitment of the bone marrow-derived cells for differentiation into or establishment of new vessels and the determining signals are governed by IL-4^28,29^.

Macrophages are recruited to damaged tissues. Macrophages can be divided into at least two major phenotypes with diverse functions: classically activated M1, driven by Th1 cytokines, and alternatively activated M2 macrophages, driven by Th2 cytokines like IL-4, IL-10 and IL-13^29^. Multiple cell types secrete type 2 cytokines in response to IL-33 and IL-33 promotes the polarization of alternatively activated M2 macrophages^30,31^. On one hand, a study investigating macrophage polarization in the macular retina and choroid has observed a higher M1 to M2 chemokine transcript ratio in advanced stage AMD compared to non-AMD subjects^32^. Consistent with this, the M2 type cytokine, IL-4, became the regulatory phenotype to suppress the choroidal neovascularization (CNV) formation^29,33^. On the other hand, another study has reported that M1 macrophages inhibit CNV while M2 macrophages enhanced it^34^.

In our study, serum levels of the cytokines IL-13 and IL-33 were also evaluated. We found no differences in serum levels of IL-13 between the MPN and AMD groups. The serum IL-33 levels between the MPNd and MPNn was not significant, however the level of IL-33 in the PVd group was significantly higher than in the PVn group. In regard to comparisons of IL-33 serum levels between MPNd and iAMD, we found a significant difference. Serum IL-33 levels showed no statistically significant difference between the MPNn, iAMD and nAMD groups. A study by Xi et al. found increased levels of IL-33 in AMD lesion areas in the retinas compared with non-AMD controls^35^. This might indicate a similar serum level of IL-33 in all the MPN and AMD groups.

Our study has some limitations. The study was observational and accordingly not providing any causality. Further, there was a difference in MPN diagnosis and duration of disease, which might influence the retinal findings, and the MPN subgroups PMF and ET were small to divide into groups with drusen and with normal retinas.

To summarize, our comparative studies of inflammatory cytokines in drusen/AMD and MPNs have shown serum levels of IL-4 and IL-33 to be elevated in patients with MPNd compared to MPNn, which may indicate that these cytokines play a role in drusen development in MPN. Our data didn’t record a significant difference in serum levels of IL-4 but a significant difference in regard to IL-33 between the MPNd and iAMD groups. Further, our findings together with previous studies indicate that all the MPN and AMD groups have levels of IL-4 or IL-33, which are elevated compared to healthy controls. These findings represent an enhanced type 2 inflammatory response in these diseases and support the association between inflammation and drusen formation. We propose using the MPNs as a “Human Inflammation Model” on drusen development. Future studies are needed to elucidate the association between AMD and MPN and the factors eliciting drusen formation, especially potential similarities between iAMD and MPNd. These studies should also address if the systemic immunomodulating treatment for MPN, herein interferon-alpha2, JAK1-2 inhibitor treatment and/or statins which dampen chronic inflammation, might actually prohibit development and progression of drusen development in MPN and thereby also prevent the development from iAMD to nAMD.

## Methods

### Study design and participants

This cross-sectional study was conducted at Zealand University Hospital (ZUH), Roskilde, Denmark at the ophthalmology and hematology departments between July 2018 and November 2020. The participants in this study consisted of the same participants as in our recent work. The description of the methods in this study will be very similar^14^. The participants included four patient types. According to the WHO 2016 criteria^36^ and the Beckman Classification^2^, we included 35 MPN patients with drusen having early or intermediate AMD (MPNd) and 27 MPN patients with healthy retinas (MPNn), 28 patients with intermediate-stage AMD (iAMD) and 29 patients with neovascular AMD (nAMD). Each participant provided written and oral informed consent. The study was approved by the Ethics Committee, Region Zealand, Denmark, the Danish Data Protection Agency and adhered to the tenets of the Declaration of Helsinki.

Exclusion criteria were patients with other active cancer, inflammatory- or autoimmune diseases, patients receiving immunomodulating treatment (Ruxolitinib, interferon-α), CRP levels > 15, and anti-VEGF injection within the last eight weeks.

### Retinal imaging and clinical data

All participants were examined at the ophthalmology department. Following pupil dilatation with tropicamide 1%, stereoscopic 45° colour fundus photograph centred on the macula were obtained (model TRG-NW8, Topcon). The photographs were evaluated in IMAGEnet i-base version 3.25.0. For use in a previous study with the same patients, each fundus photograph was graded using a simplified version of the Wisconsin age-related maculopathy grading system (WARMGS)^12,37^ to compare our results with published estimates from three large population studies^12^. We performed OCT (SD-OCT, Heidelberg Engineering, Germany) on the patients and examined the images in Heidelberg Eye Explorer version 1.9.10.0. For the current study, we used the fundus photograph and OCT to determine AMD status according to the Beckman Classification^2^. The patients also had a fundus autofluorescence (FAF) photo taken. We used these images to help diagnose or exclude GA in the included patients. Participants answered a questionnaire about their health status, medical conditions, medications, and lifestyle.

### Blood sampling and immunoassays

Venous blood from antecubital veins were sampled from each participant. We used lithium-heparin-coated tubes for CRP analysis and isolation of plasma. Tubes with silica-act-clot-activator were used to isolate serum. Part of the blood, plasma, and serum was immediately stored at − 80 °C for immunoassays. Cytokines were quantified with multiplex immunoassays (Meso Scale Discovery, Rockville, Maryland, USA) at the Technical University of Denmark (DTU). The plates were prepared according to the manufacturer’s instructions with prior identification of the dilution factor and the manufacturer’s software was used to create a standard curve (plotting mean absorbance against protein concentration) from a standard added to each plate. The standard curve was used to determine target protein concentration. Tests were run in duplications to determine the mean concentration. The coefficient of variation (CV) was calculated as the ratio of standard deviation to the mean. Mean CV values were between 1⋅9 and7⋅3 for all assays. Plate reading was done immediately after preparation on a QuickPlex SQ120 (Meso Scale Discovery).

### Statistics

We analyzed data with RStudio version 4.1.1. (for macOS, R Studio, Boston, Massachusetts, USA). Normally distributed data are shown as mean and 95% confidence interval (CI). Non-normally distributed data are shown as median and interquartile range (IQR). The distribution of continuous variables was assessed for normality with histograms and QQ-plots. We used Wiloxon’s rank sum test, One-way analysis of variance (ANOVA) or Kruskal Wallis test for continuous variables for comparisons between groups. Pearson’s Chi-squared test or Fischer’s Exact test were used for categorical variables. Linear regression to test if the outcomes depended on age. “n” represents the number of humans in the group tested. In the footnotes of the tables, the tests used are described. Power calculations were based on similar comparative immunological studies on nAMD. This exercise ended in a sample size of 26 in each group to detect a difference in variables between groups of at least 20%, with an alpha level of 0.05 and a power of 80%. We therefore aimed for 30 in each group^38,39^. Statistical significance is defined as *p* < 0.05.

## Data Availability

The datasets used/or analysed during the current study are available from the corresponding author (K.G.), upon request.
